# The impact of market segmentation and social marketing on uptake of preventive programmes: the example of voluntary medical male circumcision. A literature review

**DOI:** 10.12688/gatesopenres.12888.1

**Published:** 2018-12-11

**Authors:** Anabel Gomez, Rebecca Loar, Andrea England Kramer

**Affiliations:** 1 AVAC, AVAC, New York City, NY, 10027, USA; 2 Independent Consultant, Independent Consultant, Austin, Texas, USA; 3 Independent Consultant, Independent Consultant, St Petersburg, Florida, USA

**Keywords:** HIV prevention, segmentation, social marketing, demand creation, innovation, human-centred design

## Abstract

**Background**: The business world has long recognized the power of defining discrete audiences within a target population. However, market segmentation’s full potential has not been applied to the public health context. While some broad elements of market segmentation (e.g., age, geography) are considered, a nuanced look at behavioural and psychographic segmentation, which could greatly enhance the possibility of lasting behaviour change, is often missing.

**Segmentation**, and the associated mindset which acknowledges the multi-dimensional differences between people, allows service providers, implementers, policymakers, and government officials to target initiatives and lead to a greater likelihood of lasting behavioural change.

This paper investigates what segmentation is, how it has been applied to voluntary medical male circumcision (VMMC), how it can be applied in development, and the challenges in both measuring and adopting segmentation as part of program design.

**Methods**: We performed a detailed search of peer-reviewed literature using PubMed, ProQuest, ScienceDirect, Google Scholar, and the abstract directories of the International AIDS Society (IAS) published between January 2015 and September 2018. We also accessed articles from business databases such as the Harvard Business Review.

**Results**: Results from a VMMC-focused intervention that successfully designed and delivered segmentation-based programs in two countries demonstrated that it is possible to adapt private sector approaches. However, within the sector of global development that is most familiar with segmentation, these efforts rarely go beyond basic demographic segments.

**Conclusions**: Existing published material tends not to measure the impact of segmentation itself, but the impact of the intervention to which segmentation was applied, which makes it challenging for the development sector to invest in the approach without evidence that it works. Nonetheless, the experiences of segmentation and demand creation for VMMC do highlight the opportunity for better integrating this approach in HIV prevention and in global development and measurement initiatives.

## List of abbreviations

AGYW        Adolescent girls and young women

EAMC        Early adolescent male circumcision

EIMC         Early infant male circumcision

HIV            Human immunodeficiency virus

IAS            International AIDS Society

PrEP          Pre-exposure prophylaxis

VMMC      Voluntary medical male circumcision

## Introduction

Defining discrete audiences within a target population is a marketing approach used widely in the commercial world, where strong understanding of a consumer segment is directly tied to profits. Even private sector giants have had massive failures due to poor consumer understanding: Coca-Cola’s C2 drink is an example. In order for companies to make targeted marketing decisions they rely on segmentation, the process of dividing “a market into smaller groups of buyers with distinct needs, characteristics, or behaviors who might require separate products or marketing mixes” ^1^. Most commonly, markets are segmented by geographic, demographic, psychographic (psychological attributes such as values, attitudes, and beliefs), and behavioural factors ^2^. The resulting breakout can then be used to make strategic decisions about whom to reach and how to connect meaningfully with them through product and service experiences. A specific market segment includes individuals with similar preferences and characteristics, and different market segments are clearly differentiated ( Table 1 presents and discusses characteristics associated with useful market segments) so that the campaigns, products, and marketing tools applied to them can be implemented without overlap. Moreover, a set of criteria are typically used to define a segment – to identify individuals who share those characteristics – and those who do not fit that segment’s criteria fall into a different segment. The value of these segments is to have clear characteristics associated with a set of marketing approaches and, in turn, to drive quantifiable outcomes ^3^.

**Table 1.** Characteristics of useful market segments.

1) Identifiable/Differentiable. Customers in each segment should possess measurable key attributes – such as usage and consumption behaviours and purchasing preferences – that clearly distinguish them from customers in other segments.
2) Substantial. According to Harvard Business Review’s Gavett, “It’s usually not cost-effective to target small segments – a segment, therefore, must be large enough to be potentially profitable.” In Gichuru’s words, “Marketing segments must be large enough to meet the financial needs of the company and the product.”
3) Accessible. Are communication and distribution channels in place to reach each segment? To be useful, segments must be accessible through promotional tools.
4) Sustainable. Gavett states that “a segment should be stable enough for a long enough period of time to be marketed to strategically.” This points away from basing segmentation on attributes that tend to fluctuate, such as lifestyle.

***Sources:*** https://hbr.org/2014/07/what-you-need-to-know-about-segmentation

http://ijecm.co.uk/ ISSN 2348 0386

https://www.iiste.org/Journals/index.php/EJBM/article/viewFile/647/540

While some broad elements of segmentation, such as age and geography, have been applied in the development sector, the power of behavioural and psychographic segmentation has been largely overlooked. According to Samuel, “psychographics, which measure customers’ attitudes and interests rather than ‘objective’ demographic criteria, can provide deep insight that complements what we learn from demographics” (see Harvard Business Review article on psychographics). This type of segmentation provides a deeper understanding of the desires, needs, and decision-making considerations of a potential user of a product or service. Applied correctly, it could enhance efficacy of public health initiatives, ensure new products reach the people most likely to need and use them, and increase the likelihood of lasting behavioural change. Using the example of voluntary medical male circumcision (VMMC), this paper shows how segmentation has been applied in development and discusses the challenges in both measuring and adopting segmentation as part of program design.

In an era of constrained funding for HIV primary prevention basics like demand creation for male and female condoms, there is a need to be more targeted with resources in order to reach the right people with the right intervention – a higher priority than reaching all people. Groups sharing common attributes are more likely to respond similarly to a given demand creation strategy, but addressing all men aged between 15–19 as if they are identical is unlikely to result in cost-efficient, relevant, or relatable communication, whether it takes place at an interpersonal (e.g., peer educator) or mass level.

## Methods

To better understand what the literature reveals about the barriers to and benefits of segmentation, both for VMMC in particular and for demand creation for HIV preventive interventions more broadly, we queried scholarly databases, health and innovation journals, and other publications for studies, analyses, and peer-reviewed articles on segmentation published between January 2015 and September 2018. The types of literature considered included case studies, systematic reviews, meta-analysis, and journal articles. We performed a detailed search of the existing peer-reviewed literature using PubMed, ProQuest, ScienceDirect, Google Scholar, and the abstract directories of the International AIDS Society (IAS). We searched these sources for keywords including HIV prevention, segmentation, demand creation, demand generation, innovation, behaviours, human-centred design, segmentation evaluation, campaign qualitative evaluation, campaign success, campaign failure, target audience, social marketing, campaign lessons learned, and VMMC. Our primary application of interest was VMMC, with a secondary emphasis on HIV prevention programs related to adolescent girls and young women (AGYW). Sub-Saharan Africa was the priority region of interest, with examples elsewhere in low and middle-income countries a subordinate focus. The hundreds of results generated by these criteria were then individually examined and collated by subject-matter experts for relevance to different main aspects of this particular piece of work; full citations were developed for 61 select articles, along with complete summaries of their significance. A second round of keyword searching was conducted, this time expanding sources queried from the listed databases to include premier marketing journals and publications in order to provide more complete information regarding existing applications of segmentation (as an approach predominantly employed and evaluated predominantly within the context of private sector work). The resulting, updated bibliography included 40 sources and was reviewed a second time to produce the comprehensive list of sources (44) which informed initial drafting of the literature review text. After subsequent rounds of writing and editing, including extracting information deemed unnecessary and as a result eliminating some citations, this literature review’s current bibliography has been updated to represent its use of 37 carefully curated sources. It should be noted that this literature review did not include a need to address consent issues.

## Results

Our findings indicate that market segmentation has, to date, most often been applied to global health fields that function for profit and, as such, tend to view their clients as customers, with preferences to discover and cater to, rather than as patients with needs that are often assumed to be homogenous within a broadly defined population, such as by diagnosis or perceived need. The latter view is associated with interventions designed from the top down. However, areas of global health with a development focus or non-profit structure and culture have been slower to adopt or apply market segmentation. Moreover, existing published materials on its applications tend not to measure the impact of segmentation itself, but the impact of the intervention to which segmentation was applied, generally through uptake measures.

On top of the traditional difficulties of measuring impact in the global health and development space (e.g., long observation times, difficult data gathering, complex influences on decision-making), segmentation requires additional thought to isolate the impact of the strategies themselves. While we are able to observe that using segmentation strategies offers a framework to design more nuanced and resonant interventions, it is difficult to isolate the impact of segmentation from the many other steps, methodologies, and strategies that are part of a robust human-centred design process. As multi-disciplinary consortiums take on multi-year projects, it becomes increasingly difficult to isolate specific contributions when evaluating overall efficacy.

It is also difficult to measure impact because a segmentation strategy is not a product or a message or an experience on its own; it is a vehicle for developing them. In this way, the segmentation step is not tested directly, but indirectly through the things it produces – making it challenging to draw an objective cause and effect relationship.

Because the value and impact are difficult to isolate, segmentation is often overlooked or attempted unsystematically. Sgaier *et al.* determined that approaches to demand generation were inconsistent, not evidence-based, and poorly coordinated. This work goes on to say that “political and social factors, including ignorance of the need for strategic demand generation, may contribute to inadequate funding and focus.” The authors found that there was scant evidence on approaches to demand generation for VMMC, both in terms of understanding drivers of demand and in terms of evaluating existing interventions ^4^.

In a later article for the Stanford Social Innovation Review, Sgaier *et al*. expanded on these issues, citing particular cases. For example, in Niger, a limited understanding of the value of segmentation at the highest levels caused discussions with governments and partners to drag on for more than three years before segmentation could begin to be implemented. Even once segmentation is underway, Sgaier *et al*. note myriad issues, including restricted ability to design the research, limited number of people with experience in the segmentation process, and difficulty transferring the findings into large-scale programs ^2^. In the case of HIV prevention, the psychographic measures of risk perception and belief about the efficacy of a particular intervention proved to be a more effective approach to the segmentation of men into target audiences than basic demographic distinctions. Designing a program of social promotion that is tailored for groups based on these values, attitudes, and decision-making requirements creates the greatest likelihood for change in HIV-related behaviours among each segment ^5^.

Meanwhile, Terris-Prestholt and Windmeijer looked at interventions that promote behaviour change. They determined that the impact of interventions aimed at the ongoing behaviours that are relevant to prevention are slow to take hold and should therefore be evaluated over a longer timeframe ^6^. However, current funding mechanisms generally do not allow sufficient time for such change to take place and be observed, making interventions based on segmentation hard to evaluate.

**Figure 1.**
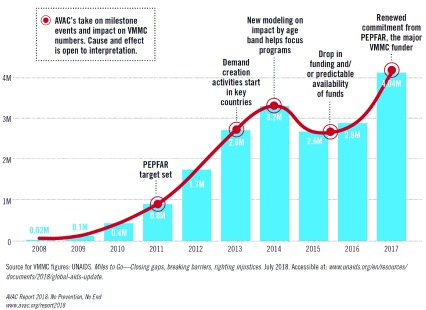
Annual number of voluntary medical male circumcisions, 2008–2017. *Source:* https://www.avac.org/sites/default/files/resource-files/AVACreport2018.pdf. *This graphic has been reproduced with permission from AIDS Vaccine Advocacy Coalition (AVAC).*

Though there are difficulties in exact measurement, the most compelling evidence for the efficacy of segmentation is to look at when and how it is used. Specifically, it is often brought in when nothing else is working or when there is a drop-off in the efficacy of an intervention. This typically occurs when generalized, homogenous efforts have reached the most easily persuaded among the target audience and nuanced interventions are needed for more resistant members of the target audience. This was the case with VMMC, where early adopters of the procedure had been reached and demand was plateauing ( Figure 1).

Segmentation can also be used to investigate why efficacy has been uneven. The knowledge gained from segmentation can be used to design specific interventions (e.g., messaging, experiences, campaigns) in situations where demand is lagging, or to prioritize outreach to specific groups if resources are constrained. This is especially true when used in conjunction with human-centred design, another methodology increasingly applied in global health programs when traditional strategies start to produce declining returns on investment.

Despite challenges, segmentation also offers a large range of opportunities for the sector. The Reproductive Health Supplies Coalition is one of a growing number of organisations in development that acknowledge that segmentation provides empirical evidence which can help guide the most efficient and effective use of resources. The Coalition states that “market segmentation data can shape family planning…(and) it can increase market efficiency for the government stewards of public resources” ^7^. It goes on to say that policymakers can use market segmentation research to “draft evidence-based policy initiatives, giving government officials a more useful context to decide which policies are worth enacting.” 

The identification of segments can also guide decisions about how to meet performance and delivery objectives within a health system. For example, segmentation studies can identify whether one group of end users will likely access a prevention service in a private clinic rather than a public one. Most obviously, segmentation can be used to guide communication at all consumer touchpoints from the community to the clinic, and on a mass scale. 

### The application in VMMC programs

The example of VMMC lends itself well to a segmentation approach. The audience of those who may undergo the procedure already encompasses distinct subdivisions by age (e.g., early infant male circumcision (EIMC), early adolescent male circumcision (EAMC), “catch-up” population segments comprised of older men). The literature is replete with examples of such demographic segmentation and its applicability to developing more effective promotions, including via social marketing ^8– 13^. Notable examples include the Kingdom of Eswatini’s successful VMMC program, which prioritised EIMC as the “sustainment” component of a comprehensive set of VMMC interventions for multiple age brackets ^14^; Lane *et al.*’s supplement of nine studies across South Africa, Zimbabwe, and Tanzania, which targeted 10–14-year old adolescent males through messaging around key incentives or barriers to VMMC uptake (motivation, counselling, wound healing, parental involvement, female peer support, quality of in-service communication, and providers' perceptions) ^15^; and an observational prospective intervention study in the Orange Farm township of South Africa, which successfully obtained male circumcision prevalence of 80% among adult men within just three months ^16^. Modelling investigations likewise find age to be a beneficial, and in some cases particularly cost-effective ^17, 18^, basis for market segmentation ^19, 20^. Geographic segmentation is similarly common ^13, 17– 19, 21^, and through modern mapping technology can offer novel applications ^21, 22^.

**Figure 2.**
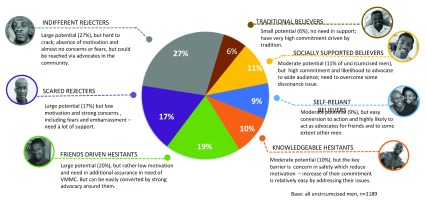
Separation of surveyed Zambian men into seven voluntary medical male circumcisions (VMMC)-candidate segments. *Source:* https://healthcommcapacity.org/wp-content/uploads/2017/06/Albert-Machinda-Society-for-Family-Health.pdf *This graphic has been reproduced with permission from The Bill and Melinda Gates Foundation.*

Importantly, however, recent work also emphasizes the need for segmentation that goes beyond age to distinguish among individuals’ perceived motivations or disincentives for VMMC, as well as beyond the candidate population for VMMC to highlight the role of decision-making “influencers” (i.e., female partners, family members like parents, grandparents, and parents-in-law, trusted community leaders like sports team coaches), who may be effectively targeted through social marketing promotions of VMMC to encourage its uptake among the men in their lives ^23^. Figure 2 depicts a representation of segments applied to VMMC in Zambia.

The work to segment Zambian and Zimbabwean men along behavioural and psychographic lines provides the most straightforward example of a non-age-based population dissection with findings readily applicable to social marketing interventions. In this case, men were segmented in alignment with factors that motivated and/or supported them on a personal level to undergo VMMC. Men were also segmented according to influences at the community or structural level that discouraged or encouraged uptake ^24^. Another important finding from the “influencer” cluster of studies relates to how campaigns are conducted *after* segmentation. VMMC candidates, perhaps due to the intimate nature of the decision and procedure, exhibit a strong preference for individual over mass communication on this issue ^9, 25^. This preference is borne out by both the insignificant results yielded in a study which leveraged the mass communication platform of SMS to deploy VMMC-related information and counselling ^26^, as well as by two unsuccessful VMMC promotion case studies which cited a lack of consideration for sociocultural context (such as including the perspectives and gaining the support of “traditional leaders, healers and circumcisers”) as a reason for failure ^27^. In demand creation campaigns, personal counselling or one-to-one approaches favourably impacted willingness to undergo VMMC or to consider it for one’s dependents. Quality market segmentation conducted before beginning such an individualised intervention has the potential to make this otherwise costly and labour-intensive – yet highly effective – approach more feasible to implement ^9, 16, 28^.

Market segmentation can also identify whom *not* to target. A 2015 analysis “explored correlates of male circumcision status among men and their social, economic, health and sexual behaviour factors.” This analysis provided characteristics for better targeting and intervention design. In this case, limited resources for uptake campaigns could be directed toward populations of greatest need and minimize the use of resources directed at segments that were unlikely to choose VMMC under any circumstance ^29^.

There is also some evidence for market segmentation’s value in creating demand for HIV preventive services outside of VMMC. Cremin *et al*. use a mathematical model to isolate certain subsets of Nairobi’s population in which HIV incidence is on the rise, in contrast to its trend of decline at the city level, and to suggest optimal interventions for reducing HIV infection among these high-risk groups ^30^. Reed *et al*. extrapolate lessons learned from VMMC scale-up in the region to support oral pre-exposure prophylaxis (PrEP) expansion among another targeted market segment ^31^. Also addressing the AGYW audience, Celum *et al*. explore how social marketing and innovative market segmentation can increase demand for and optimise uptake and effective use of PrEP ^32^; Eakle *et al*. point to this potent combination as a particularly useful means of enhancing PrEP demand generation among more sceptical communities ^33^; and Luecke *et al*. examine the demographic and behavioural correlates of preferred PrEP formulations, arguing that a deeper understanding of women’s product preferences can guide not only product development but also drive demand creation through social marketing ^34^. Sgaier, too, opines from family planning work in India that psychographic-behavioural segmentation can better forecast demand and, “as in the private sector, a staged market launch that actively stimulates uptake can be used to match appropriate products to suitable customers” (see devex article on contraception in Uttar Pradesh women). Ayikwa and Jager advocate for social marketing as the “ultimate weapon” in combating HIV/AIDS transmission and overcoming related stigmas ^35^.

All these papers corroborate Rao and McCoy, who state that “behaviour change isn’t just about crafting the perfect message; creating better programs requires really listening to and understanding the patient experience” [ https://ssir.org/articles/entry/fostering_behavior_change_for_better_health#bio-footer]. They stress that “borrowing tools from the private sector…to understand, track, and influence customers can greatly enhance global health programs that require changes in attitudes or behaviour.”

## Discussion / Recommendations

The literature collectively drive toward the idea that what the public health sphere needs is a new mindset. Instead of viewing its target audience as patients with diagnoses, the audience should be seen as multidimensional consumers with preferences and needs. Current public health segmentation has been almost solely demographic; though valuable at a basic level, this is rudimentary ^36^. Research marketing pioneer Daniel Yankelovich states in the Harvard Business Review that demographic segmentation “implies that differences in reasons for buying, in brand choice influences, in frequency of use, or in susceptibility will be reflected in differences in age, sex, income, and geographical location. But this is usually not true” (see Harvard Business Review article on market segmentation). Basically, age segmenting can only be useful as a very general indication of patterns of behaviour, as not everyone in the same age band will behave the same way or respond the same way to experiences. Even “geodemographic classifications such as ACORN (a classification of regional neighborhoods), while useful for indicating likely very general patterns of spending power, do not reveal the absurd assumption that everyone…drives the same car, reads the same newspapers, eats the same food and so on” (see The Marketing Journal article on market segmentation). Demographic and age segmentation are some of the easiest to develop, but they provide limited guidance. In order to successfully effect change, implementers need to connect deeply with their audience by looking past its superficial characteristics to the attitudinal, behavioural, and contextual factors that guide its members’ decision-making. This does not mean that public health solutions should be fragmented, but rather that precise messaging – still framed in the broader context of health goals – can be developed for and directed to audiences with whom it is most likely to resonate strongly ^4^.

## Conclusions

At this stage, it is not yet possible to definitively conclude that market segmentation leads to measurably better HIV prevention results, but it *can* be asserted that market segmentation leads to *interventions* with measurably better HIV prevention results. The literature present ample evidence for the value of market segmentation as a component of demand creation for HIV prevention interventions, including VMMC and, more recently, oral PrEP. While traditional applications of market segmentation in healthcare, such as age and geography, remain useful as components of more nuanced population stratifications, behavioural-psychographic segmentation presents the greatest potential for efficacy in uptake of HIV prevention measures, both broadly and in the case of VMMC specifically. In a later article, also for the Harvard Business Review, Yankelovich and Meer write that “non-demographic segmentation began more than 40 years ago as a way to focus on the differences amongst customers that matter most strategically” ^37^. Ultimately, what the public health sphere needs is a shift: from considering possible users of its products as a single group with the same desires and behaviours based on age or location to seeing them as multidimensional consumers with individual preferences and needs.

## Data availability

*All data underlying the results are available as part of the article and no additional source data are required*
