# Supplementary material for: The impact of market segmentation and social marketing on uptake of preventive programmes: the example of voluntary medical male circumcision. A literature review
Source: Gates Open Res. 2018 Dec 11;2:68. [Version 1] doi: 10.12688/gatesopenres.12888.1 (PMC6480503; doi:10.12688/gatesopenres.12888.1)
Supplement: Peer review history

# Peer review: Reviewer response for version 1

**DOI:** 10.21956/gatesopenres.13982.r26814

**Published:** 2019-03-19

**Authors:** Jason B. Reed, Manya Dotson, Sema Sgaier

**Affiliations:** 1 Jhpiego, Baltimore, MD, USA; 2 Surgo Foundation, Washington, D.C., USA; 3 Harvard T.H. Chan School of Public Health, Boston, MA, USA; 4 University of Washington, Seattle, WA, USA

The referee team finds the article to be an important contribution to the field because it reinforces the potential value of an underappreciated approach to global development/non-profit public health programming, specifically related to program uptake. A case is made that the private sector is better at market segmentation. Development/public health donors often prescribe demographic and behavioral sub-populations—sex, age, geographic area, wealth quintile—for program services based upon epidemiology and return on investment expectations. Implementers may simply resort to the same or similar sub-populations for structuring demand creation campaigns. Going beyond these conventional public health categories to include psychographic and behavioral segments may represent an important opportunity for greater cost-effectiveness in program execution, by more efficiently influencing uptake. However, the authors stop short of describing how the private sector (more) successfully determines psycho-behavioral market segments and acts upon them in selection of messages and channels. This may be because little is published on methodologies from the private sector, which has little incentive to share proprietary approaches. This may well be beyond the scope of this paper, but some discussion of bases of segmentation could be helpful to the reader to understand the concept. As it is, the reader is left with the vague feeling that “everyone is different” and therefore all messaging must be micro-tailored, which would be even less efficient/cost-effective than existing approaches.

 The article could be improved by defining terminology upfront, e.g., demographic, geographic, behavioral vs. psychographic market segmentation. A strong definition of segmentation, including the four qualities of viable segments, is provided here for further consideration:

 “Heterogeneous markets are divided into homogenous markets by identifying what are known as the appropriate bases of segmentation. An appropriate segmentation base might be age in a

 market where young people prefer a different product or service than old people. Sex, socioeconomic status, and residence are common segmentation bases due to assumptions about differing preferences between males and females, the rich and the poor, and the urban and rural. Many other segmentation bases exist, including psycho-graphics and lifestyle. In marketing, the appropriateness of one or more segmentation bases is evaluated in terms of whether each basis is identifiable (able to be measured), actionable (theoretically able to be changed through an intervention), accessible (defines populations able to be reached), responsive (empirically are changed by an intervention), substantial (large in size), and stable (do not change for a period long enough to design, monitor and evaluate an intervention)” Frank *et al.* (1972 ^1^).

 The details of parameters for the literature review are clear. The number of professional disciplines of subject matter experts reviewing and summarizing the findings is less clear, though presumably comprised of some or all of the authorship team. Given the disconnect between private and public sector approaches to segmentation, it would be valuable to know whether interpretations of relevance and importance (to impact and as specifically relates to voluntary medical male circumcision) varied by background of the experts.

 The main conclusions appear to be framed in relation to public health intervention uptake (or adherence). However, the other a priori interests stemming from market segmentation that may be of equal (or arguably even greater) importance may take precedence, e.g., using segmentation to identify services/products more aligned with potential users preferences, demand forecasting, and preference of where to access products and services (public vs. private sector, formal vs. informal outlets). If market segmentation were more thoughtfully used earlier in processes, segmentation might not be so frequently the approach of last resort. In turn, judging segmentation impact might be broader than solely focused on improvements in uptake.

We have read this submission. We believe that we have an appropriate level of expertise to confirm that it is of an acceptable scientific standard.

## References

[1] FrankREMassyWFWindY: Market Segmentation. *Prentice-Hall*.1972;

# Peer review: Reviewer response for version 1

**DOI:** 10.21956/gatesopenres.13982.r26900

**Published:** 2019-01-29

**Authors:** Steve Kretschmer

**Affiliations:** 1 DesireLine LLC, Istanbul, Turkey

1. The work cites the current literature adequately. Further clarification on the following points, as detailed in the comments I have provided in the paper itself, will strengthen the clarity and accuracy of the paper. An annotated version of the article can be downloaded here.
  - Clarify the forms of market segmentation throughout the paper to be specific about which forms of segmentation are being discussed in each point made on its use
  - Clarify segmentation as a means to dividing the market and identifying the differences to then target differential interventions/services/products vs. as a method itself to understand and intervene.
2. The study design is appropriate and the work is technically sound
3. Some additional details of methods and analysis need clarification to allow replication by others
4. Statistical analyses not applicable
5. All source data are available
6. The conclusions drawn are adequately supported by the results

I have read this submission. I believe that I have an appropriate level of expertise to confirm that it is of an acceptable scientific standard.

# Peer review: Reviewer response for version 1

**DOI:** 10.21956/gatesopenres.13982.r26815

**Published:** 2019-01-04

**Authors:** Beth Skorochod

**Affiliations:** 1 CollaborateUp, Washington, DC, USA

The title includes both segmentation and social marketing but social marketing is not defined in the article and is not discussed at any length. I would remove social marketing from the title, or have it play a bigger role in the article (and define it upfront or in a table as has been done with segmentation).

 While the authors state that segmentation was not measured extensively, there are too few examples for any reader to try to replicate any work done with segmentation. The work of VMMC, which is used as the main case study for the piece, includes insufficient detail for replication and does not include specific data for Zimbabwe or Zambia. How did behavioral segmentation impact uptake of VMMC in these two countries? What were the challenges of executing against the segmentation results. What was the impact of activities across each of the 7 segments in Zambia? Can additional data on impact and/or details on execution be included for these two countries? Even the VMMC studies mentioned don't seem to point specifically to the examples in Zambia and Zimbabwe.

 The definitions of quality market segmentation in this paper are too vague, making the prospect of applying it to a program very difficult. Table 1 provides characteristics of useful market segments but additional detail or examples are needed. Table 1 states that segments should be substantial enough to be profitable or to meet the financial needs of a company. While this works for the commercial sector, it is unhelpful for development work which is typically not about profitability or even cost effectiveness. Can authors include examples of a 'substantial' size that would be applicable and relevant to HIV prevention? In addition, "sustainable" is also used to describe a useful market segment, stating that the segment should be stable enough for a long-enough period of time to be marketed strategically and warns off using something like "lifestyle' which too easily fluctuates. But what is a long-enough period of time to be marketed strategically? Do attitudes and behaviors shift less frequently than 'lifestyle? If not using age or demographics, what type of attributes should be used that will not fluctuate easily and how long do those attributes need to be stable? Better defining or providing concrete examples from public health segmentation (or the commercial sector) will strengthen the piece and make it more relevant to implementers.

 The article seems at times to conflate demand generation and demand creation with segmentation. In paragraph 5 of the Results section concludes that segmentation is often overlooked or unsystematic, using the Sgaier article's criticism of poor demand creation approaches as evidence, but these seem two different things. Demand creation does not necessarily include behavioral segmentation. While it may include demographic segmentation, that is not the focus of the piece. TheTerris-Prestholt and Windmeijer article on behavior change interventions is also cited as a reason why segmentation would be hard to evaluate and measure, but the conclusion seems forced unless segmentation is part of the reason behavior change interventions take a longer time to take hold.

I have read this submission. I believe that I have an appropriate level of expertise to confirm that it is of an acceptable scientific standard, however I have significant reservations, as outlined above.
